# Histologic healing and factors associated with complete remission following conventional treatment in ulcerative colitis

**DOI:** 10.1177/17562848221140659

**Published:** 2022-12-06

**Authors:** Øyvind Steinsbø, Arne Carlsen, Ole Gunnar Aasprong, Lars Aabakken, Espen Tvedt-Gundersen, Steinar Bjørkhaug, Rune Gjerde, Lars Normann Karlsen, Tore Grimstad

**Affiliations:** Department of Medical Gastroenterology, Stavanger University Hospital, Gerd-Ragna Bloch Thorsens gate 8, Stavanger, 4068, Norway; Department of Medical Gastroenterology, Stavanger University Hospital, Stavanger, Norway; Department of Pathology, Stavanger University Hospital, Stavanger, Norway; Department of Medical Gastroenterology, Rikshospitalet University Hospital, Oslo, Norway; Department of Medical Gastroenterology, Stavanger University Hospital, Stavanger, Norway; Department of Medical Gastroenterology, Stavanger University Hospital, Stavanger, Norway; Department of Medical Gastroenterology, Stavanger University Hospital, Stavanger, Norway; Department of Medical Gastroenterology, Stavanger University Hospital, Stavanger, Norway; Department of Medical Gastroenterology, Stavanger University Hospital, Stavanger, Norway; Department of Clinical Science, University of Bergen, Bergen, Norway

**Keywords:** 5-ASA, corticosteroids, endoscopy, histology, remission, risk factors, ulcerative colitis

## Abstract

**Background:**

Endoscopic and histological activity scores in ulcerative colitis (UC) are associated with clinical outcomes and have become important targets of clinical trials. However, these endpoints have been scarcely investigated in patients receiving only conventional treatment.

**Objective:**

We aimed to assess the deep and complete remission rates after 3 months of conventional treatment in patients with newly diagnosed UC with moderate to severe endoscopic activity. We also aimed to investigate whether selected clinical and biochemical variables at baseline were associated with complete remission status after 3 months.

**Design:**

This was a prospective cohort study.

**Methods:**

Newly diagnosed patients with active UC commencing 5-aminosalicylate, corticosteroid, and/or azathioprine treatment were consecutively included. Clinical, biochemical, endoscopic, and histological data were collected at baseline and after 3 months. Rates of *clinical remission* (Partial Mayo Score ⩽ 2), *mucosal healing* (Mayo Endoscopic Score ⩽ 1), and *histologic healing* (Nancy Index ⩽ 1) were determined. *Deep remission* was assessed as clinical remission plus mucosal healing and *complete remission* as deep remission plus histologic healing. Predictors of complete remission were identified by logistic regression.

**Results:**

A total of 180 patients were included in the study. Deep remission and complete remission occurred in 62.8% and 42.2% of patients, respectively. Thus, of patients in deep remission one-third had persistent histologic activity. Histologic activity in mucosally healed patients was associated with higher symptom scores and faecal calprotectin levels. Of baseline variables, less endoscopic distribution and disease activity showed strongest association with achieving complete remission, and limited distribution in combination with moderate activity gave highest odds for complete remission (odds ratio: 4.1, 95% confidence interval: 7.69–2.18).

**Conclusion:**

In patients with mucosal healing, persistent histologic activity was a common finding and was associated with increased disease activity. Pancolitis and severe inflammatory activity at baseline were associated with lower complete remission rates.

## Introduction

Ulcerative colitis (UC) is a chronic inflammatory bowel disease (IBD) in which the clinical course can vary over time, ranging from a quiescent to fulminant disease state with serious complications and disability.^1^ Medical treatment is guided by disease activity, and the treatment targets in American and European guidelines are clinical remission, characterized by the absence of faecal blood and diarrhoea or by a Partial Mayo Score (PMS) ⩽ 2, and mucosal healing based on a Mayo Endoscopic Score (MES) ⩽ 1.^2,3^ The achievement of both clinical remission and mucosal healing has been termed ‘deep remission’ and is associated with subsequent long-term clinical remission and lower risk of colectomy.^4–6^

Persistent histologically active inflammation has been associated with increased risk of relapse, recurrent hospitalization, and risk of colorectal neoplasia.^7–10^ Of note, patients with mucosal healing may still have persistent histologic activity.^10,11^ These observations call into question whether histologic healing, which is often defined by the absence of acute inflammation,^12^ should be a treatment target in addition to clinical remission and mucosal healing. This state may be termed ‘complete remission’.^13^

Many patients diagnosed with moderate to severe UC receive first-line treatment with conventional therapeutics, such as 5-aminosalisylate (5-ASA), corticosteroids, and/or immunomodulators, including azathioprine, but are ultimately switched to treatment with biologics over time.^14^ Therefore, it would be of interest to study the prevalence of deep and complete remission achieved by first-line conventional treatment, as these treatment targets are predictive of the clinical outcome and can guide the medical management.

In this study, we aimed to assess the deep and complete remission rates after 3 months of conventional treatment in patients with newly diagnosed UC with moderate to severe endoscopic activity. We also investigated whether selected clinical and biochemical variables at baseline are associated with the patient’s complete remission status after 3 months.

## Materials and methods

### Patients

In this observational cohort study, previously untreated patients aged ⩾16 years who were newly diagnosed with UC were consecutively recruited from the Department of Gastroenterology, Stavanger University Hospital Norway, from 04/01/12 to 01/03/19. The diagnosis of UC was based on a combined clinical, laboratory, endoscopic, and histological evaluation according to the European Crohn’s and Colitis Organization (ECCO) guidelines.^15^ Inclusion criteria were active UC and commencement of conventional treatment after the first study visit. Exclusion criteria were failure to fulfil diagnostic criteria for UC, pregnancy, inability to consent, inability to adhere to the study protocol, inactive disease, receiving biological treatment during the study, or missing endoscopic or histological evaluation of disease activity at the visits. The patients were included during a study visit at diagnosis (V0) and re-evaluated after 3 months of treatment (V3). Identical data were registered at both study visits. The study was registered at ClinicalTrials.gov (NCT01551563).

### Conventional treatment regimen

The patients received conventional treatment according to the ECCO guidelines,^2^ defined as 5-ASA, corticosteroids, and/or immunomodulators, such as azathioprine. 5-ASA and corticosteroids were administered orally and/or topically.

### Disease activity

#### Symptom score

Symptoms of disease activity were assessed by the PMS, which is a clinical scoring index based on the Mayo score.^16^ The Mayo score consists of a clinical (PMS) and endoscopic (MES) disease activity rating in which the clinical assessment consists of three items: stool frequency, stool blood content, and physicians’ general estimation of disease activity. Each item is rated on a 4-point scale from 0 to 3; thus, the PMS ranges from 0 to 9 points. A PMS ⩽2 was defined as clinical remission in accordance with previous studies.^17^

#### Biomarkers

Serum C-reactive protein (CRP), haemoglobin (Hb), albumin (±3 days), and faecal calprotectin (from 4 weeks before to 3 days after inclusion visit, and ±7 days after last visit) were analysed at both visits.

### Endoscopic assessment

A colonoscopy was performed at both visits to assess the distribution and severity of the mucosal inflammation. The disease distribution was defined according to the Montreal classification:^18^ E1, ulcerative proctitis; E2, left-sided UC; and E3, extensive UC.

The severity of the mucosal inflammation was rated according to the MES^16^ by the endoscopist without central reading. The MES consists of the endoscopic activity item of the Mayo score and is rated on a 4-point scale from 0 to 3: 0, normal mucosa (without signs of inflammation); 1, mild mucosal inflammation (mild friability, reduced vascular pattern, mucosal erythema inflammation); 2, moderate inflammation (friability, erosions, complete loss of vascular pattern, and significant erythema); and 3, severe inflammation (ulcerations and spontaneous bleeding). The mucosal area with the most severe inflammation throughout the colon determined the MES. Active UC was defined as MES ⩾ 2 and mucosal healing as MES ⩽ 1.

#### Histologic assessment

During colonoscopy, a minimum of two biopsies were taken from right and left part of the colon, and from the rectum. The biopsies were formalin-fixed, paraffin-embedded, sectioned, and stained with haematoxylin and eosin. Histologic inflammation was evaluated in biopsies from colorectum (right colon biopsies missing for seven patients at V0 and 23 patients at V3, left colon biopsies missing for 9 patients at V3).

Histologic inflammation was determined by a senior pathologist and graded from 0 to 4 according to the Nancy Index (NI), based on ulcers, acute inflammatory infiltrates, and chronic inflammatory infiltrates^19^ as: 0, no histological significant disease; 1, chronic inflammatory infiltrate; 2, mildly active disease with acute inflammatory cells (presence of neutrophils); 3, moderate active disease with acute inflammatory cells; and 4, severely active disease with ulceration.

At baseline, the NI was defined as the colorectal segment with the highest score. Histologic healing was assessed after 3 months, and defined as NI ⩽ 1 in all biopsies from all of the colorectal segments available. Histologic activity was defined as NI ⩾ 2 in at least one colorectal segment.

### Deep remission and complete remission

Deep remission was defined as PMS ⩽ 2 and MES ⩽ 1, whereas complete remission was defined as deep remission plus histologic healing.

### Statistical analysis

Normal distribution was assessed by the Shapiro–Wilk test. For normally distributed data, t-tests were used. For data that were not normally distributed and rank values, the Wilcoxon test was used for paired groups and Mann–Whitney test for unpaired groups. Chi-square test was used for binominal data.

Correlations were analysed using Pearson’s r test for normally distributed data and Spearman’s r for categorical variables and data that were not normally distributed. The Spearman’s rank correlation coefficient (*r*_s_) was regarded as negligible (0–0.2), weak (0.2–0.4), moderate (0.4–0.6), strong (0.6–0.8), or very strong (0.8–1).

When developing regression models, univariable binary logistic regression analyses were performed using complete remission as the dependent variable. Age, Hb, albumin, CRP, and faecal calprotectin were used as numeric, independent variables. Sex, disease distribution, PMS, MES, NI, and an exploratory composite category combining disease distribution and MES were used as categorical, independent variables. If the dataset of a numeric variable ranged ⩾10^2^, as it was for CRP and faecal calprotectin, the Log_10_ of the value was used in the analysis. If a group variable contained a group with fewer than 5 individuals, this group was merged with the neighbouring group (i.e. NI = 0 was merged with NI = 1).

The odds ratio (OR) with 95% confidence interval (CI) was reported for all logistic regression analyses, reflecting the shift in odds by an increase of 1 for numeric variables and a 10-fold increase when the Log_10_ of the value was used, or the difference in odds from a reference category of the categorical variables.

Backward stepwise selection models were then fitted to exclude non-significant independent variables by the likelihood-ratio test. Variables with *p* < 0.2 in the univariable logistic regression analyses were included in the multivariable models, and the maximum number of variables per model was defined by 1/10 of the sample size of the binary dependent variable (complete remission). The variables with the lowest *p* values were included in the first model. The statistically significant variables from the first model were included in subsequent models.

*p* Values < 0.05 were considered significant for all statistical analyses, which were performed in SPSS 26.0 (IBM SPSS Statistics for Windows, Version 26.0. Armonk, NY, USA).

The reporting of this study conforms to the STROBE statement.^20^

## Results

### Patients and baseline characteristics

**Figure 1.**
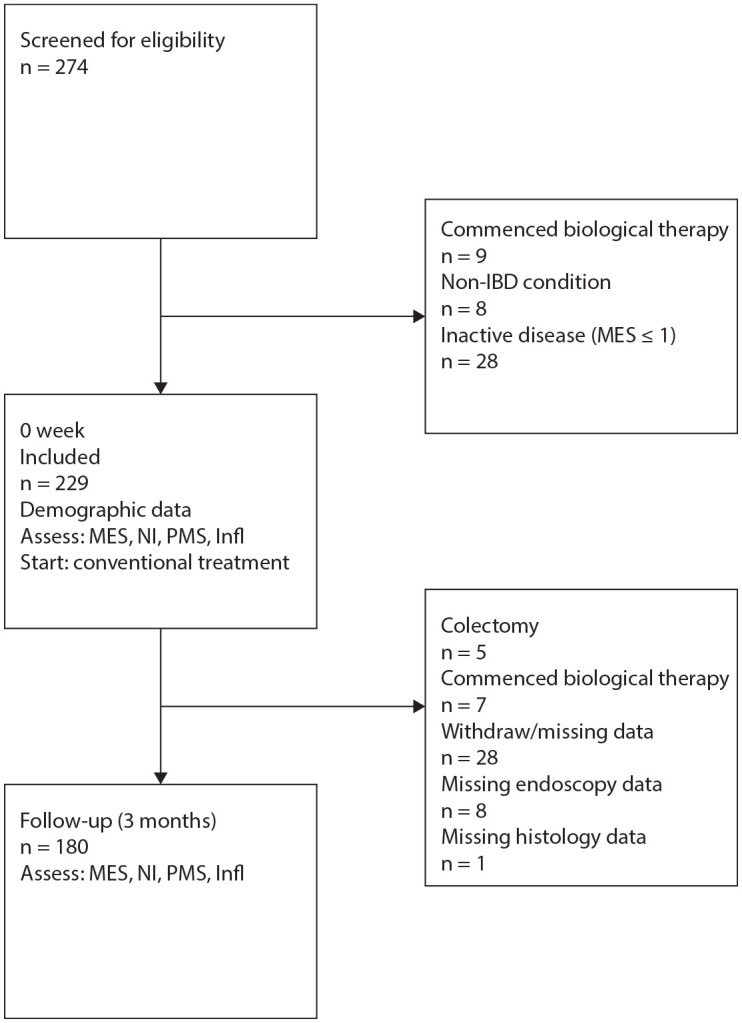
Flow chart showing study recruitment and reasons for exclusion. Infl, inflammatory markers in blood and faeces; MES, Mayo Endoscopic Score; NI, Nancy Index; PMS, Partial Mayo Score.

Of 274 eligible patients, 229 fulfilled the inclusion criteria and 180 patients completed the study (Figure 1). Baseline characteristics, including the medications prescribed at inclusion, are summarized in Table 1. At the start of the study, 51 patients (28.3%) were diagnosed with proctitis, 59 (32.8%) with left-sided disease, and 70 (38.9%) with pancolitis. At inclusion, 179 patients started treatment with 5-ASA and 73 patients were given corticosteroid treatment (72 patients in combination with 5-ASA and 1 patient commenced corticosteroids in monotherapy). Two patients also started on azathioprine (in combination with 5-ASA and corticosteroids). Further details on medical treatment at V0 and V3 is shown in Supplemental Table 1.

**Table 1.** Baseline characteristics in 180 patients with UC.

Variable		
Age (years)	34 (25–49)	
Sex M/F	101/79 (56.1/43.9)	
CRP (mg/L), *n* = 179	6.3 (2.2–21)	
Albumin (g/L), *n* = 179	39.6 (36–43)	
Haemoglobin (g/dL)	13.8 (12.8–14.8)	
Faecal calprotectin (mg/kg), *n* = 160	880 (315–1896)	
PMS	5 (3–7)	
MES	2 (2–3)	
Total Mayo Score	7 (5–9)	
NI	3 (2–3)	
Disease distribution		
Rectum (E1)	51 (28.3)	
Left colon (E2)	59 (32.8)	
Pan colon (E3)	70 (38.9)	
Medication started at diagnosis		
5-ASA	179 (99.4)	Dose daily (mg)
Oral only	*54* (*30.0*)	4800 (3200–4800)
Topical only	*23* (*12.8*)	1000 (1000–2000)
Oral + topical	*102* (*56.7*)	5800 (5000–6400)
Corticosteroid	73 (40.6)	Dose, start (mg)*
Prednisolone	*54* (*30.0*)	30 (30–30)
Budesonide	*2* (*1.1*)	9 (9–9)
Methylprednisolone	*17* (*9.4*)	40 (40–60)
Azathioprine	2 (1.1)	100 (50–150)

Data are given as median (IQR) or *n* (%) unless otherwise noted.

* For corticosteroids, the daily dose was typically tapered by 5 mg weekly.

5-ASA, 5-aminosalisylate; CRP, C-reactive protein; IQR, interquartile range; MES, Mayo Endoscopic Score; NI, Nancy Index; PMS, Partial Mayo Score; UC, ulcerative colitis.

### Disease activity at baseline and after 3 months

**Figure 2.**
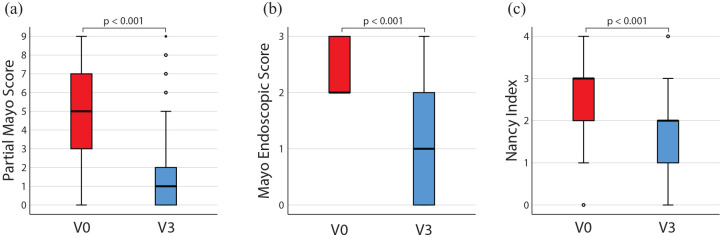
Disease activity in patients with UC at baseline (V0) and after 3 months (V3) assessed by (a) PMS, (b) MES, and (c) NI. Wilcoxon test, *p* < 0.001 for all three differences. MES, Mayo Endoscopic Score; NI, Nancy Index; PMS, Partial Mayo Score; UC, ulcerative colitis.

The median PMS decreased significantly, from 5 (interquartile range: 3–7) at V0 to 1 (0–2) at V3 (p < 0.001). Similarly, the median MES decreased from 2 (2–3) to 1 (0–2) (*p* < 0.001) and NI from 3 (2–3) to 2 (0–2) (*p* < 0.001, Figure 2).

### Remission and healing rates after 3 months

Clinical remission (PMS ⩽ 2) was reported by 138 patients (76.7%). Mucosal healing (MES ⩽ 1) was achieved in 133 patients (73.9%) and histologic healing (NI ⩽ 1 in all colorectal segments available) in 87 patients (48.3%). In all, 83 of the mucosally healed patients (62.4%) achieved both mucosal healing and histologic healing. Thus, persistent histologic activity was observed in 50 (37.6%) of the patients with mucosal healing.

Deep remission (clinical remission and mucosal healing) was observed in 113 patients (62.8%) and complete remission (deep remission and histologic healing) in 76 patients (42.2%). Thus, 37 (32.7%) patients achieving deep remission had persistent histologically active inflammation.

**Figure 3.**
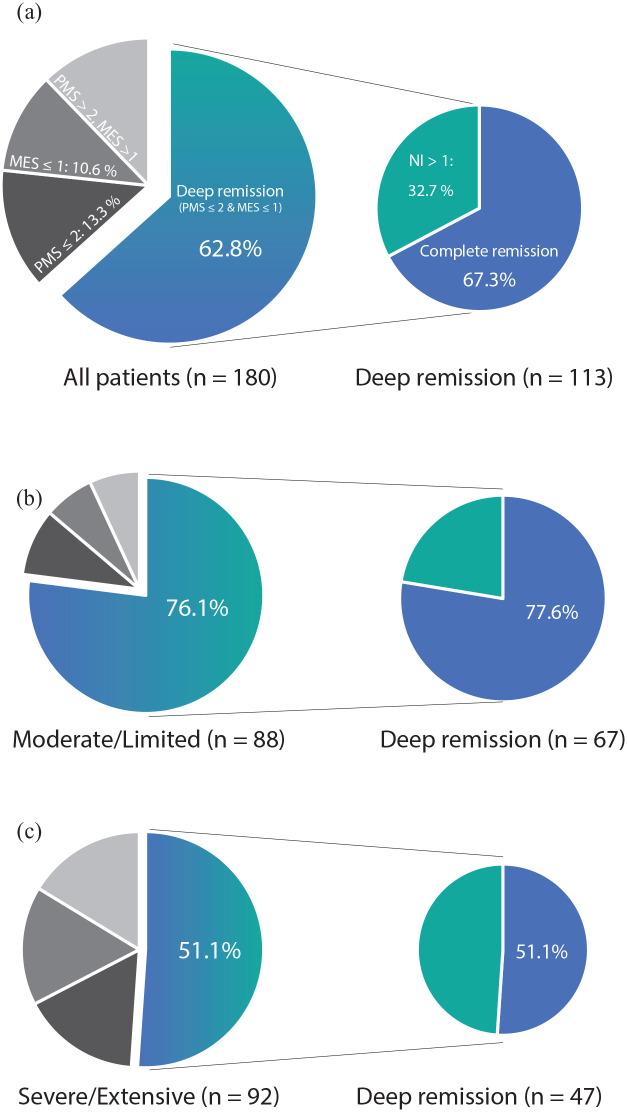
Deep remission rates (left) and histologic healing rates in the group with deep remission (right) after 3 months of conventional treatment in (a) all patients and in patients grouped by baseline endoscopic findings; (b) ‘Moderate/Limited’, MES 2 and proctitis or left-sided colitis; (c) ‘Severe/Extensive’, MES 3 or pancolitis. PMS, Partial Mayo Score; MES, Mayo Endoscopic Score, NI, Nancy Index.

Remission and healing rates are depicted in Figure 3(a).

### PMS and faecal calprotectin in patients with mucosal and histologic healing

Of the 133 patients with mucosal healing, 55 (41.4%) had an MES of 0 and 78 (58.6%) had an MES of 1. The patients with an MES of 0 had significantly lower PMSs and faecal calprotectin levels than the patients with an MES of 1 (*p* < 0.05).

**Figure 4.**
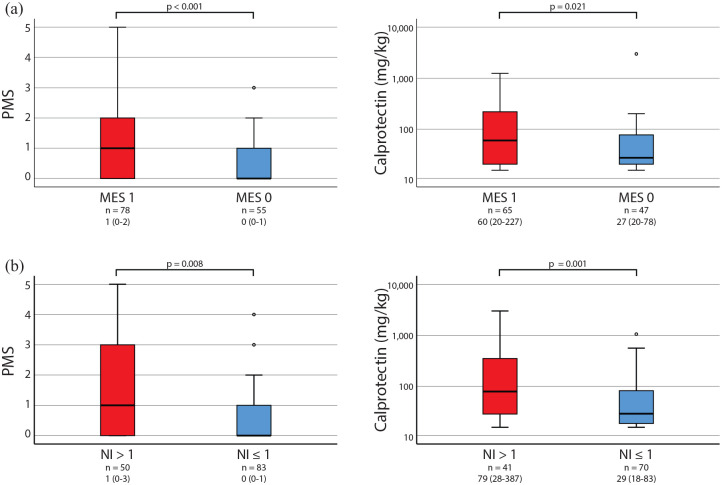
Partial Mayo Score and faecal calprotectin levels in UC patients with mucosal healing (*n* = 133). Patients were grouped by (a) MES 1 *versus* MES 0 or (b) NI > 1 (persistent acute histologic inflammation) *versus* NI ⩽ 1 (histologic healing). Patient groups were compared by the Mann–Whitney U test. Number of patients in each subgroup and median (range) values are given below each graph. PMS, Partial Mayo Score; MES, Mayo Endoscopic Score; NI, Nancy Index.

The patients with combined mucosal healing and histologic healing had significantly lower PMSs and faecal calprotectin levels than mucosally healed patients with persistent histologic activity (*p* < 0.05; Figure 4).

### Baseline variables associated with complete remission after 3 months

#### Univariable analyses

Limited disease distribution (left-sided colitis or proctitis), lower MES scores (MES = 2), and normal concentrations of CRP and albumin at baseline were all significantly associated with achieving complete remission after 3 months, whereas age, sex, PMS, haemoglobin, and faecal calprotectin were not.

Furthermore, corticosteroid treatment was associated with lower odds of achieving complete remission. Per oral 5-ASA showed close to significant lower odds of achieving complete remission as compared to combined per oral and topical 5-ASA treatment. No significant associations were observed with increasing 5-ASA dose (Table 2(a)).

**Table 2:** Logistic regression analyses in 180 patients with UC using *complete remission* after 3 months of conventional treatment as the dependent variable and baseline values for independent variables. (a)

	Dependent variable: complete remission						
Independent variables		Univariable analyses			Multivariable analyses		
	n	OR	95% CI	*p*	OR	95% CI	*p*
Gender					Ni		
Male	101	0.859	(0.474–1.558)	0.617			
Female	79	1					
Age	180	1.001	(0.982–1.022)	0.888	Ni		
Disease distribution				0.003**			0.015*
Proctitis (E1)	51	3.759	(1.744–8.104)	0.001**	3.186	(1.450–7.003)	0.004**
Left-sided colitis (E2)	60	2.153	(1.034–4.483)	0.04*	1.824	(0.857–3.882)	0.119
Pancolitis (E3)	69	1			1		
PMS				0.177	Excl		
Quiescent (PMS 0-1)	9	3.474	(0.778–15.51)	0.103			
Mild (PMS 2–4)	63	1.683	(0.795–3.562)	0.174			
Moderate (PMS 5–6)	56	0.965	(0.440–2.117)	0.929			
Severe (PMS 7–9)	52	1					
MES							
MES 2	129	3.2	(1.537–6.662)	0.002**	2.650	(1.244–5.641)	0.011*
MES 3	51	1					
NI				0.100	Excl		
NI 0–1	9	7.0	(0.969–50.6))	0.054			
NI 2	75	1.750	(0.485–6.314)	0.393			
NI 3	84	1.111	(0.309–3.998)	0.872			
NI 4	12	1					
CRP (Log_10_)	179	0.511	(0.301–0.868)	0.013*	Excl		
Calprotectin (Log_10_)	160	0.614	(0.331–1.138)	0.121	Excl		
Albumin	179	1.104	(1.036–1.177)	0.002**	Excl		
Haemoglobin	180	1.176	(0.967–1.430)	0.103	Excl		
5-ASA formula	179			0.032*	Excl		
Topical	23	1.894	(0.752–4.770)	0.175			
Per oral	54	0.513	(0.254–1.035)	0.062			
Topical and per oral	102	1					
5-ASA total dose (g)	177	0.897	(0.762–1.056)	0.191	Ni		
Corticosteroids							
No	107	2.134	(1.145–3.976)	0.017*	Excl		
Yes	73	1					

**Table:** (b)

		Dependent variable: complete remission			Disease group
Composite variables	n	OR	95% CI	*p*	
MES 2/proctitis	42	6.0	(2.06–17.479)	0.001**	‘Moderate/Limited’
MES 2/left-sided colitis	46	3.273	(1.165–9.192)	0.024*	
MES 2/pancolitis	41	1.241	(0.418–3.686)	0.697	‘Severe/Extensive’
MES 3/proctitis	9	0.857	(0.143–5.130)	0.866	
MES 3/left-sided colitis	14	0.818	(0.176–3.804)	0.798	
MES 3/pancolitis	28	1			

**Table:** (c)

		Dependent variable: complete remission		
Disease group	*n*	OR	95% CI	*p*
‘Moderate/Limited’	88	4.1	(7.69–2.18)	<0.001**
‘Severe/Extensive’	92	1		

5-ASA, 5-aminosalisylate; CI, confidence interval; CRP, C-reactive protein; Excl, excluded from the multivariable analysis due to p > 0.05 in multivariable models; MES, Mayo Endoscopic Score; Ni, not included in multivariable analysis due to *p* > 0.2 in univariable analyses; OR, odds ratio; PMS, Partial Mayo Score; UC, ulcerative colitis.

* *p* < 0.05, **p < 0.005.

#### Multivariable analysis

Limited disease distribution and lower MES at the start of the study had the strongest association with complete remission (Table 2(a)).

### Remission rates in patients with lower MES and limited disease distribution

MES and disease distribution correlated significantly (Spearman’s *r*: 0.214, *p* = 0.004), and the patients were categorized accordingly. In univariable analysis, only lower MES (2 *versus* 3) in combination with limited distribution (proctitis and left-sided colitis *versus* pancolitis) showed a significant positive association with complete remission (Table 2(b)).

The patients were allocated to ‘Moderate/Limited’ (MES = 2 and limited distribution, *n* = 88) and ‘Severe/Extensive’ (MES = 3 or pancolitis, *n* = 92) according to baseline data. When comparing the Moderate/Limited group to the Severe/Extensive group, the deep remission rates were significantly higher (75.2% *versus* 51.1%, *p* = 0.001), as were the complete remission rates (59.1% *versus* 26.1%, *p* < 0.001; Table 2(c) and Figure 3(b) and (c)).

## Discussion

The main findings in our study were that 63% of patients diagnosed with UC and moderate to severe endoscopic disease activity achieved deep remission after 3 months of conventional therapy, and 42% were classified as complete remission. The PMSs and faecal calprotectin levels were significantly lower in patients with combined mucosal and histologic healing compared to patients with mucosal healing but persistent histologically active inflammation. Moreover, moderate endoscopic inflammation and non-extensive disease distribution at baseline were associated with greater occurrence of deep and complete remission.

The deep remission rate found in our study cannot be directly compared to previous studies, as ‘deep remission’ has not been consistently defined. However, earlier studies of the effect of 5-ASA, corticosteroids, or azathioprine have reported mucosal healing rates of 25–77%, 31–52%, and 53–68%, respectively,^21^ which are in line with our findings.

Approximately two-thirds of the patients in deep remission also achieved complete remission, whereas one-third had persistent histologically active inflammation. Similar discrepancies between endoscopic and histologic findings have been reported by others.^10,11,22^ A comparison of PMSs and faecal calprotectin levels between these two groups indicated that the patients with histologic healing had lower disease activity. Notably, histologic healing in addition to mucosal healing has been associated with long-term clinical remission.^7^ Thus, the discrepancy between deep and complete remission rates underscores the significance of histologic evaluation in patients with mild or quiescent endoscopic findings. Accordingly, the Selecting Therapeutic Targets in Inflammatory Bowel Disease II initiative from the International Organization for the Study of IBD recently acknowledged histologic evaluation as an important factor in assessing the depth of treatment response.^23^ Of note, the histologic healing rate is biased by the histologic scoring index (and cut-off), and in a recent study NI ⩽ 1 gave lower healing rates than Geboes score ⩽ 3.1 and Robarts histologic index ⩽ 6.^22^ Less is known about how the number of biopsy sites may impact on the healing rates.^24^ In some previous studies, the histological examination has been confined to distal colorectal segments.^25–27^ In this study, we included proximal segments, in accordance with many of the observational studies reporting beneficial effect of histologic healing.^8,10,28^ Notably, in our cohort 18 of 113 patients in deep remission scored NI ⩽ 1 in rectal biopsies and NI ⩾ 2 in proximal colonic segments (data not shown). Whether these patients have the same disease course as patients in complete remission is unclear and is planned to be assessed in a follow-up study.

We also found that a stricter endoscopic definition of mucosal healing (MES of 0 *versus* 1) was associated with lower disease activity, as shown by lower PMS and faecal calprotectin levels in our study. Several large clinical trials have previously regarded both MES = 0 and MES = 1 as indicative of mucosal healing.^29,30^ However, more recent data demonstrated a reduced relapse rate with an MES of 0 compared to an MES of 1.^31^ The latter is in line with our findings, suggesting that complete endoscopic normalization (MES = 0) may represent the optimal endoscopic treatment target.

The baseline endoscopic score and disease distribution showed the strongest associations with complete remission in our study. The literature on baseline variables that predict the short-term response to conventional therapeutics is limited, except for corticosteroid treatment of acute severe colitis. In these patients, combined clinical and biochemical scores have outperformed endoscopic scores in predicting therapeutic failure.^32^ In two long-term follow-up studies of 5-ASA treatment, extensive disease and severe endoscopic activity at inclusion were associated with higher therapeutic requirements,^33^ and extensive disease distribution was associated with increased risk of clinical relapse.^34^

The endoscopic activity score and disease distribution were intercorrelated. To investigate the relevance of this finding, we developed a composite categorical variable. We found that patients with less endoscopic activity in combination with limited distribution achieved the highest complete remission rates. As most endoscopic activity scores do not consider the extent of the inflammation, composite scores based on the product of endoscopic activity and disease distribution have been developed.^35–37^ These composite scores have been reported to exhibit a stronger association with treatment failure and clinical relapse than endoscopic activity scores.^38–40^ Our findings are in line with these reports, except that severe endoscopic activity was associated with lower remission rates in our study regardless of disease distribution. Whether our findings are related to slower healing in patients with severe endoscopic activity or these patients have poorer outcomes independent of disease distribution should be evaluated in a longitudinal follow-up study.

The level of conventional treatment showed no significant association with complete remission in the multivariable regression. Corticosteroids were more frequently started in patients with severe endoscopic findings at V0 (Supplemental Table 1), and the negative association with complete remission was not significant when adjusting for MES and disease extent (Table 2(a)). We conclude that severe endoscopic activity and extensive disease represented poor prognostic factors despite intensified conventional treatment in these patients.

Two patients started azathioprine treatment at inclusion. Despite not representing the standard therapeutic strategy at diagnosis and that 3 months may be too short to show effect of this drug, these cases reflected clinical practice and were included. However, azathioprine was not included in the logistic analysis because of the low number and because of the reasons mentioned above.

This study has limitations. Patients commencing biological treatment during the study period (*n* = 16) were excluded. They expectedly had severe disease activity or did not respond sufficiently to conventional treatment within the study period. This may have influenced the results. Whether 3 months is sufficient to achieve histologic healing is, to the best of our knowledge, not known and should be further investigated in a longitudinal study. In addition, the definitions of deep and complete remission lack standardization, which makes it difficult to compare our results with previous findings. The study design was observational, which has some inherent limitations. The endoscopic activity was scored by the endoscopist(s) without central reading, and the NI was assessed by a single pathologist. On the other hand, this may also be one of the strengths of this study design, as it reflects the relevance of combining clinical, endoscopic, and histological evaluation in a clinical setting. The cohort in this study is also relatively large compared to previous studies. Moreover, we present relevant data on conventional treatment that can be compared to trials reporting on the mucosal and histologic healing rates of new therapeutics, and we use a histologic index that has been recommended used in observational studies and clinical practice.^41^

In summary, 3 months of conventional treatment of active UC induced deep remission in 63% of incident cases in our clinic, of which one-third had persistent histologically active inflammation. In a subgroup analysis of patients with mucosal healing, endoscopic complete normalization or adjunct histologic healing was associated with lower clinical and biochemical disease activity. Moderate disease activity combined with non-extensive disease distribution at diagnosis was associated with increased complete remission rates. Our findings support the relevance of histological evaluation in patients with endoscopically quiescent to mild disease and suggest that baseline endoscopic activity and disease distribution should be included in the risk assessment.

## Supplemental Material

sj-docx-1-tag-10.1177_17562848221140659 – Supplemental material for Histologic healing and factors associated with complete remission following conventional treatment in ulcerative colitis Supplemental material, sj-docx-1-tag-10.1177_17562848221140659 for Histologic healing and factors associated with complete remission following conventional treatment in ulcerative colitis by Øyvind Steinsbø, Arne Carlsen, Ole Gunnar Aasprong, Lars Aabakken, Espen Tvedt-Gundersen, Steinar Bjørkhaug, Rune Gjerde, Lars Normann Karlsen and Tore Grimstad in Therapeutic Advances in Gastroenterology

## Declarations

**Ethics approval and consent to participate:** This study was approved by the regional ethics committee (REC 2011/2631) and conducted according to the principles expressed in the Declaration of Helsinki. All patients provided written informed consent to participate in the study.

**Consent for publication:** All patients gave their consent to publish their own de-identified data in the study.

**Availability of data and materials:** The data underlying this article will be shared on reasonable request to the corresponding author.
