# Supplementary material for: Histologic healing and factors associated with complete remission following conventional treatment in ulcerative colitis
Source: Ther Adv Gastroenterol. 2022 Dec 6;15:17562848221140659. doi: 10.1177/17562848221140659 (PMC9729989; doi:10.1177/17562848221140659)
Supplement: sj-docx-1-tag-10.1177_17562848221140659 – Supplemental material for Histologic healing and factors associated with complete remission following conventional treatment in ulcerative colitis [file sj-docx-1-tag-10.1177_17562848221140659.docx]

# Supplementary table 1.

Legend: Medical treatment at V0 and V3, and endoscopic findings at V0 and complete remission rate at V3 in patients grouped by medical treatment started at V0.

|  | | Treatment V3 | | | | | | | Severe / Extensive^4^  endoscopic findings V0 | Complete remission V3 |
| --- | --- | --- | --- | --- | --- | --- | --- | --- | --- | --- |
|  |  | No treatment  (n = 9) | 5-ASA monotherapy  (n = 129) | Corticosteroids^2^ started at V3  (n = 26) | Corticosteroids^2^ started before V3^3^  (n = 9) | 5-ASA + azathioprine  (n = 3) | Biologic drugs  (n = 3) | Missing data  (n = 1) |  |  |
| Treatment V0 | 5-ASA monotherapy  (n = 107) | 9 | 80 | 15 | 2 | 1 | 0 | 0 | 32  (30.0%) | 53  (49.5%) |
|  | Corticosteroids^1^  (n = 73) | 0 | 49 | 11 | 7 | 2 | 3 | 1 | 60  (82.2%) | 23  (31.5%) |

^1^ Corticosteroids (n = 73): monotherapy (n = 1), combination with 5-ASA (n = 70) or combined with 5-ASA and azathioprine (n = 2)

^2^ Corticosteroids in combination with 5-ASA ± azathioprine.

^3^ Prolonged corticosteroid treatment started at V0, or new treatment started at some point between V0 and V3

^4^ Severe/extensive = MES 3 or pancolitis
